# Interaction between plasma phospholipid odd-chain fatty acids and GAD65 autoantibodies on the incidence of adult-onset diabetes: the EPIC-InterAct case–cohort study

**DOI:** 10.1007/s00125-023-05948-x

**Published:** 2023-06-10

**Authors:** Anna-Maria Lampousi, Sofia Carlsson, Josefin E. Löfvenborg, Natalia Cabrera-Castro, María-Dolores Chirlaque, Guy Fagherazzi, Paul W. Franks, Christiane S. Hampe, Paula Jakszyn, Albert Koulman, Cecilie Kyrø, Conchi Moreno-Iribas, Peter M. Nilsson, Salvatore Panico, Keren Papier, Yvonne T. van der Schouw, Matthias B. Schulze, Elisabete Weiderpass, Raul Zamora-Ros, Nita G. Forouhi, Stephen J. Sharp, Olov Rolandsson, Nicholas J. Wareham

**Affiliations:** 1 Institute of Environmental Medicine, Karolinska Institutet, Stockholm, Sweden; 2 Department of Risk and Benefit Assessment, Swedish Food Agency, Uppsala, Sweden; 3 Department of Epidemiology, Regional Health Council, IMIB-Arrixaca, Murcia, Spain; 4 Consortium for Biomedical Research in Epidemiology and Public Health (CIBERESP), Madrid, Spain; 5 Department of Health and Social Sciences, Murcia University, Murcia, Spain; 6 Deep Digital Phenotyping Research Unit, Department of Precision Health, Luxembourg Institute of Health, Strassen, Luxembourg; 7 Department of Clinical Sciences, Clinical Research Center, Skåne University Hospital, Lund University, Malmö, Sweden; 8 Department of Public Health and Clinical Medicine, Family Medicine, Umeå University, Umeå, Sweden; 9 Department of Medicine, University of Washington School of Medicine, Seattle, WA USA; 10 Unit of Nutrition and Cancer, Cancer Epidemiology Research Program, Catalan Institute of Oncology (ICO-IDIBELL), Barcelona, Spain; 11 Blanquerna School of Health Sciences, Ramon Llull University, Barcelona, Spain; 12 Medical Research Council Epidemiology Unit, Institute of Metabolic Science, University of Cambridge School of Clinical Medicine, Cambridge, UK; 13 National Institute for Health Research Biomedical Research Centre Core Nutritional Biomarker Laboratory, University of Cambridge School of Clinical Medicine, Cambridge, UK; 14 Danish Cancer Society Research Center, Copenhagen, Denmark; 15 Navarra Public Health Institute, Pamplona, Spain; 16 Navarra Institute for Health Research (IdiSNA), Pamplona, Spain; 17 Dipartimento di Medicina Clinica e Chirurgia, Federico II University, Naples, Italy; 18 Cancer Epidemiology Unit, Nuffield Department of Population Health, University of Oxford, Oxford, UK; 19 Julius Center for Health Sciences and Primary Care, University Medical Center Utrecht, Utrecht University, Utrecht, the Netherlands; 20 Department of Molecular Epidemiology, German Institute of Human Nutrition Potsdam-Rehbruecke, Nuthetal, Germany; 21 German Center for Diabetes Research (DZD), Neuherberg, Germany; 22 Institute of Nutritional Science, University of Potsdam, Nuthetal, Germany; 23 International Agency for Research on Cancer, World Health Organization, Lyon, France

**Keywords:** Diabetes, GAD65Ab, Heptadecanoic, Islet autoimmunity, OCFA, Pentadecanoic

## Abstract

**Aims/hypothesis:**

Islet autoimmunity may progress to adult-onset diabetes. We investigated whether circulating odd-chain fatty acids (OCFA) 15:0 and 17:0, which are inversely associated with type 2 diabetes, interact with autoantibodies against GAD65 (GAD65Ab) on the incidence of adult-onset diabetes.

**Methods:**

We used the European EPIC-InterAct case–cohort study including 11,124 incident adult-onset diabetes cases and a subcohort of 14,866 randomly selected individuals. Adjusted Prentice-weighted Cox regression estimated HRs and 95% CIs of diabetes in relation to 1 SD lower plasma phospholipid 15:0 and/or 17:0 concentrations or their main contributor, dairy intake, among GAD65Ab-negative and -positive individuals. Interactions between tertiles of OCFA and GAD65Ab status were estimated by proportion attributable to interaction (AP).

**Results:**

Low concentrations of OCFA, particularly 17:0, were associated with a higher incidence of adult-onset diabetes in both GAD65Ab-negative (HR 1.55 [95% CI 1.48, 1.64]) and GAD65Ab-positive (HR 1.69 [95% CI 1.34, 2.13]) individuals. The combination of low 17:0 and high GAD65Ab positivity vs high 17:0 and GAD65Ab negativity conferred an HR of 7.51 (95% CI 4.83, 11.69), with evidence of additive interaction (AP 0.25 [95% CI 0.05, 0.45]). Low dairy intake was not associated with diabetes incidence in either GAD65Ab-negative (HR 0.98 [95% CI 0.94, 1.02]) or GAD65Ab-positive individuals (HR 0.97 [95% CI 0.79, 1.18]).

**Conclusions/interpretation:**

Low plasma phospholipid 17:0 concentrations may promote the progression from GAD65Ab positivity to adult-onset diabetes.

**Graphical Abstract:**

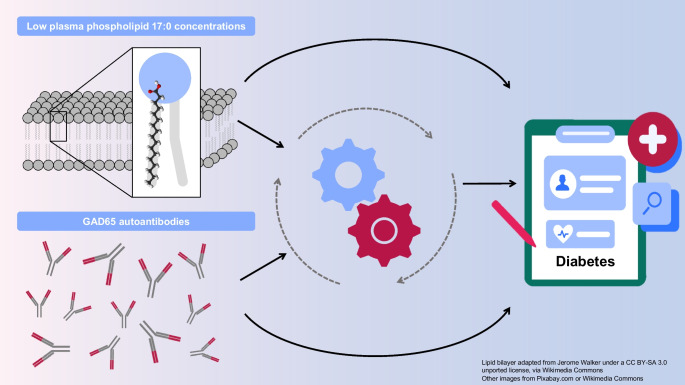

**Supplementary Information:**

The online version of this article (10.1007/s00125-023-05948-x) contains peer-reviewed but unedited supplementary material.


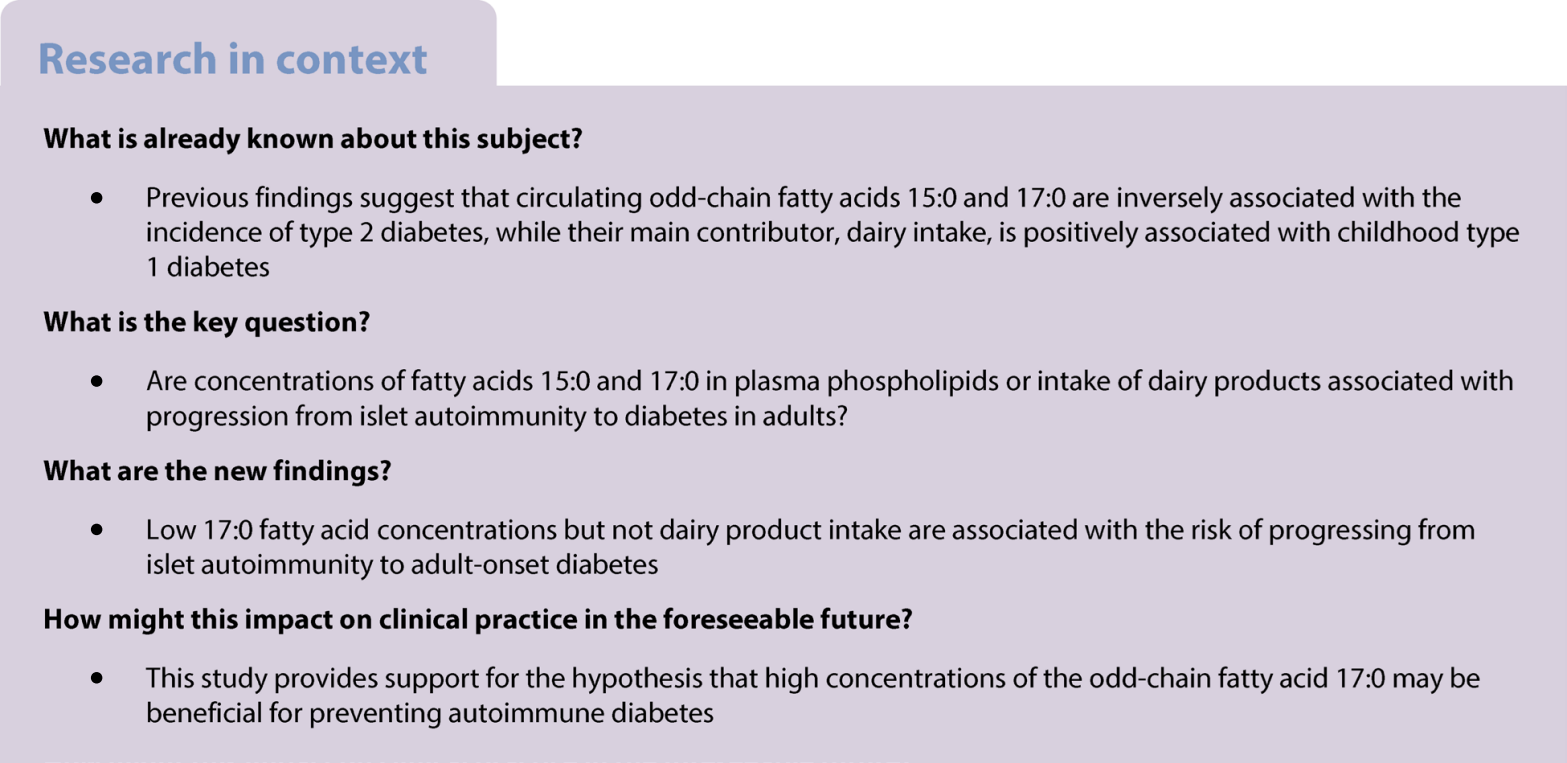


## Introduction

The presence of autoantibodies against one or more islet cell autoantigens is an indicator of destruction in the insulin-producing beta cells and usually precedes the diagnosis of type 1 diabetes [1, 2]. Autoantibodies targeting GAD65 (GAD65Ab) have additionally been linked to an increased incidence of adult-onset diabetes with no evident autoimmune phenotype [3]. Strategies for preventing or postponing progression from islet autoimmunity to overt diabetes are needed but, so far, risk factors are largely unknown [4].

Circulating odd-chain fatty acids (OCFA) pentadecanoic acid (15:0) and heptadecanoic acid (17:0) are associated with favourable metabolic properties [5] and a decreased risk of type 2 diabetes [6]. In line with these findings, intake of dairy products (the main contributor to OCFA concentrations [7]), particularly fermented products such as yoghurt, is linked to a reduced risk of type 2 diabetes [8]. On the other hand, a high intake of dairy products may increase the risk of type 1 diabetes in children [4]. This is possibly attributed to the strong antigenicity of cow’s milk and the molecular mimicry between cow’s milk proteins and proteins in the islets of Langerhans [9]. Regarding the risk of autoimmune diabetes with adult-onset, such as type 1 diabetes or latent autoimmune diabetes in adults (LADA), a potential association with OCFA concentrations or dairy product intake has not been addressed.

Our aim was to examine whether plasma phospholipid concentrations of 15:0 and/or 17:0 or dairy product intake are associated with progression from GAD65Ab positivity to adult-onset diabetes and to evaluate the potential interaction between these fatty acids and GAD65Ab status on the incidence of adult-onset diabetes.

## Methods

### Study population

**Fig. 1:**
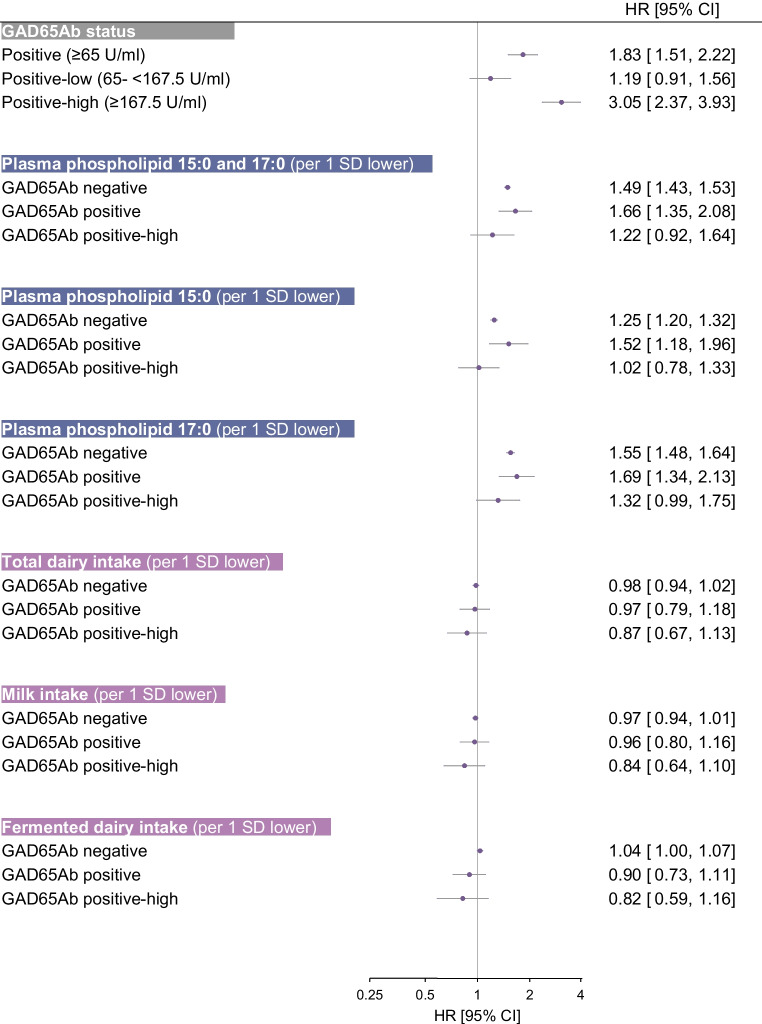
HR (95% CI) of incident diabetes in relation to GAD65Ab positivity vs negativity and per 1 SD lower plasma phospholipid 15:0 and/or 17:0 concentrations or dairy product intake, stratified by GAD65Ab status (GAD65Ab negative [*n* cases=10,735, *n* non-cases=13,900], GAD65Ab positive [*n* cases=389, *n* non-cases=274] and GAD65Ab positive-high [*n* cases=223, *n* non-cases=104]. HRs were adjusted for age (underlying time scale), centre (stratified baseline hazard), sex, education level, smoking status, physical activity and BMI. HRs for fatty acids or intake of dairy products were additionally adjusted for total energy intake and intake of alcohol, fruits, vegetables, cereal and cereal products, fish and shellfish, and red and processed meat (g/day)

We used data from the EPIC-InterAct study, which is nested within eight of the ten participating countries of the European Prospective Investigation into Cancer and Nutrition (EPIC) study. EPIC-InterAct was created with the aim of studying risk factors for type 2 diabetes and has a prospective case–cohort design, previously described in detail [10]. Briefly, the EPIC study recruited 340,234 individuals across 26 centres in Denmark, France, Germany, Italy, the Netherlands, Spain, Sweden and the UK, between 1991 and 1998. The cohort was followed until 31 December 2007, date of death or date of diabetes diagnosis, whichever occurred first. This generated 3.99 million person-years of follow-up (median 10.9 years), during which 12,403 incident cases of type 2 diabetes were verified and included as cases in the EPIC-InterAct case–cohort study. From the full cohort, a random, centre-stratified subcohort of 16,835 participants (4.9% of the entire cohort) was also assembled. After exclusion of individuals with prevalent diabetes, unknown diabetes status, or diabetes after censoring (*n*=681), 16,154 participants remained in the subcohort and were included in the case–cohort. By design [10], 778 of these participants overlap with the incident type 2 diabetes cases. This is an efficient design as exposure information only needs to be assessed for the cases and the subcohort, while inferences can be drawn for the full cohort. From our analytical sample we excluded individuals without information on plasma phospholipid OCFA (*n*=483), GAD65Ab status (*n*=352) or important covariates (i.e. education, smoking, physical activity and BMI) (*n*=929) and excluded individuals in the top or bottom 1% of the ratio of energy intake to basal metabolic rate (*n*=717). This left 11,124 cases and 14,866 subcohort participants, 692 of which were cases (electronic supplementary material [ESM] Fig. 1). All participants provided written informed consent, and the study was approved by the local ethics committee in the participating countries and the Internal Review Board of the International Agency for Research on Cancer (IARC).

### Diabetes ascertainment

Case verification was based on at least two sources, including self-report, linkage to primary care registers, secondary care registers, medication use (drug registers), hospital admissions and mortality data. In Denmark and Sweden, cases were identified through local and national diabetes and pharmaceutical registers. Some of the cases might meet the criteria for LADA, which include GAD65Ab positivity, onset ≥35 years and absence of insulin requirement for 6–12 months following diagnosis [11]. This could not be verified since GAD65Ab was assessed at baseline and not at diagnosis. Thus, we will refer to the study outcome as adult-onset diabetes.

### Baseline dietary and covariate assessment

Information on habitual diet during the past year was collected through validated country/centre-specific questionnaires covering locally consumed foods [12, 13]. Total energy and nutrient intakes were calculated with the standardised EPIC Nutrient Database [14]. The consumption of dairy products was assessed as intake of total dairy products (milk, milk beverages, yoghurt, thick fermented milk, curd, cheese, cream desserts, milk-based puddings, dairy creams, milk for coffee and creamers), milk and fermented dairy products (yoghurt, thick fermented milk and cheese) in g/day.

Weight (kg), height (m) and waist circumference (cm) were measured by trained personnel in most centres, and self-reported in some parts of the UK and France. In Umeå (Sweden), waist circumference was not measured (*n*=1620). Demographic, health and lifestyle characteristics were assessed by questionnaire [13]. Self-reported physical activity was categorised according to the Cambridge physical activity index as inactive, moderately inactive, moderately active or active [15].

### Laboratory measurements

Plasma samples were drawn at baseline and stored in liquid nitrogen at the IARC in France or at local biobanks at −196°C, except in the case of Denmark (−150°C) and Umeå (−80°C). Relative concentrations of plasma phospholipid fatty acids were analysed between 2010 and 2012 at the Medical Research Council Human Nutrition Research (UK) using previously described methods [16]. Briefly, fatty acid methyl esters were obtained after hydrolysation and methylation of the fatty acids. Fatty acid identification was based on retention time in gas chromatography (J&W HP-88, 30 m length, 0.25 mm internal diameter; Agilent Technologies, Santa Clara, CA, USA) equipped with flame ionisation detection (7890N GC; Agilent Technologies). The relative concentrations measured were expressed as a percentage of total phospholipid fatty acids (mol%).

Plasma GAD65Ab levels were measured in baseline samples from EPIC-InterAct participants, using a previously described radio-binding assay method [17], and expressed in relative units according to the WHO standard [18]. The cut-off for GAD65Ab positivity was set at ≥65 U/ml (99% specificity and 85% sensitivity [3]) and the median value among GAD65Ab-positive individuals (167.5 U/ml) was used as the cut-off for high GAD65Ab positivity, as previously done in this population [19].

### Statistical analysis

All analyses were performed with Stata Statistical Software Release 16 (StataCorp, College Station, TX, USA). Baseline characteristics of the case–cohort participants were described as proportions for the categorical variables and as mean with SD or median with IQR for the continuous variables, stratified by GAD65Ab status within the subcohort and incident cases. These characteristics were compared between GAD65Ab-positive and -negative individuals based on the *p* value obtained by *χ*^2^ test for proportions, the Student’s *t* test for means, and the Kruskal–Wallis *H* test for medians. Logistic regression was used to estimate the OR of GAD65Ab positivity and high positivity in relation to tertiles of and per 1 SD increase in 15:0 and/or 17:0 concentrations and dairy product intake. The tertiles and SDs of OCFA and intake of dairy products were calculated based on the distribution in the subcohort. Correlations between OCFA, intake of dairy products and other dietary factors were assessed with Spearman rank correlation coefficients within the subcohort.

Prentice-weighted Cox regression models with age as the underlying time scale [20] were used to estimate HRs and 95% CIs of adult-onset diabetes in relation to the following factors: (1) GAD65Ab status at baseline (positive [≥65 U/ml], positive-low [65 to <167.5 U/ml], or positive-high [≥167.5 U/ml] vs negative [<65 U/ml]); and (2) 1 SD lower 15:0 and/or 17:0 or dairy product intake at baseline, stratified by GAD65Ab status. According to this method, subcohort participants contributed person-years since baseline, while non-subcohort cases were adjusted to enter the study right before their failure time [20]. All models were adjusted for age (underlying time scale), sex, education (none, primary, technical/professional, secondary, tertiary), smoking (never, former, current), physical activity (inactive, moderately inactive, moderately active, active) and BMI (kg/m^2^). Analyses with OCFA concentrations or dairy intake as exposure were additionally adjusted for total energy intake (kJ/day), and intake of alcohol, fruits, vegetables, cereal and cereal products, fish and shellfish, and red and processed meat (g/day). Baseline hazard was stratified by centre in Cox models [21]. Visual inspection of log (−log) plots for the main exposures suggested that the proportional hazards assumption was unviolated.

Using the same Cox models, we estimated HRs of diabetes in relation to combinations of GAD65Ab status and tertiles of 15:0 and/or 17:0 concentration. The stratum that conferred the lowest risk when the two exposures were considered jointly was used as the reference group [22]. Additive interaction between GAD65Ab positivity or high positivity and the lowest tertile of OCFA concentrations was estimated by the proportion attributable to interaction (AP) with 95% CIs [23]. AP represents the proportion of cases among the doubly exposed that is attributable to the interaction between the two exposures and may help identify the population subgroups that could benefit more from a targeted intervention [24]. As a complement, we assessed interactions on the multiplicative scale, by fitting product terms for GAD65Ab status and tertiles of OCFA into the Cox models. Based on these models, we derived the HRs of diabetes in relation to the lowest vs highest tertiles of OCFA within strata of GAD65Ab status, and we present the *p* value for the coefficient of the product term between OCFA and GAD65Ab positivity or high positivity vs negativity. A *p* value of <0.05 reflects effect heterogeneity of low OCFA across strata of GAD65Ab status.

### Sensitivity analyses

We further adjusted the HRs for potential confounders that were not included in the main models and had a high proportion of missing data (i.e. waist circumference [7% missing] and family history of type 2 diabetes [51% missing]), after restricting the sample to those with available information on these covariates. We also adjusted for additional dietary factors including beverages in g/day (soft drinks, coffee, tea), nutrients in mg/day (calcium, magnesium) or μg/day (vitamin D) and relative concentrations of polyunsaturated plasma phospholipid fatty acids in mol%. The analyses of 15:0 and 17:0 were mutually adjusted. To avoid reverse causation, we excluded individuals diagnosed with diabetes during the first 2 years of follow-up (*n*=1010) or who had baseline HbA_1c_ ≥48 mmol/mol (≥6.5%) (*n*=2106).

### Post hoc analyses

We additionally investigated whether potential contributors to OCFA, other than dairy product intake, are associated with progression to adult-onset diabetes. Specifically, we used the same Cox models to estimate HRs of diabetes in relation to 1 SD lower fruit, vegetable and total dietary fibre intakes, stratified by GAD65Ab status. HRs were adjusted for age (underlying time scale), centre (stratified baseline hazard), sex, education level, smoking status, physical activity, BMI, total energy intake and intake of alcohol, dairy products, cereal and cereal products, fish and shellfish, and red and processed meat (g/day). The analyses of fruit and vegetables were mutually adjusted.

## Results

### Baseline characteristics

In total, 2.0% (*n*=297) of the subcohort and 3.5% (*n*=389) of the cases were GAD65Ab positive at baseline (Table 1). Baseline characteristics did not differ by GAD65Ab status in the subcohort. Cases with GAD65Ab positivity were more likely to be women and have lower BMI, smaller waist circumference, lower energy intake, lower intake of fish and red meat, and higher concentrations of OCFA compared with GAD65Ab-negative cases. GAD65Ab positivity or high positivity was not associated with plasma phospholipid OCFA or dairy product intake in cross-sectional analyses (ESM Table 1).

**Table 1:** Baseline characteristics by GAD65Ab status among all eligible subcohort and incident diabetes cases

Characteristic	Subcohort			Incident diabetes cases		
	GAD65Ab negative	GAD65Ab positive	*p* value^a^	GAD65Ab negative	GAD65Ab positive	*p* value^b^
Individuals, *n*	14,569^c^	297^d^		10,735	389	
Follow-up, years	12 (2.3)	11.9 (2.4)	0.574	6.9 (3.3)	6.5 (3.5)	0.052
Age, years	52.2 (9.1)	52.8 (9.3)	0.330	55.5 (7.6)	55.0 (8.4)	0.213
Female sex	62.5	64.6	0.440	50.1	58.4	0.001
BMI, kg/m^2^	26.0 (4.2)	25.8 (4.2)	0.359	29.7 (4.7)	28.4 (5.1)	<0.001
Waist circumference, cm^e^	86.3 (12.6)	86.2 (12.7)	0.902	97.7 (12.3)	93.8 (13.8)	<0.001
Family history of type 2 diabetes^f^	19.0	13.5	0.097	36.3	31.1	0.124
High education	20.8	20.5	0.316	13.3	13.9	0.104
Smokers	25.8	23.2	0.459	27.8	28.0	0.513
Physically active	20.1	19.5	0.518	16.8	18.5	0.131
Energy intake, kJ/day	8941 (2653)	9012 (2774)	0.652	9121 (2820)	8632 (2531)	<0.001
Alcohol intake, g/day	6.4 (0.9–18.4)	6.0 (1.5–17.8)	0.707	6.2 (0.6–20.4)	5.4 (0.6–15.2)	0.060
Food and drink consumption, g/day						
Fruit	193.2 (103.2–316.4)	207.1 (115.4–325.2)	0.237	182.4 (96.1–316.4)	186.7 (100.4–301.1)	0.548
Vegetables	155.7 (101.2–238.5)	151.8 (97.7–224.3)	0.758	149.1 (94.8–234.4)	147.5 (97.4–218.4)	0.418
Cereal and cereal products	197.9 (140.8–273.6)	189.8 (134.4–271.2)	0.388	198.2 (138.3–273.7)	192.2 (131.4–266.4)	0.125
Total dietary fibre	21.9 (17.4–27.1)	22.1 (17.3–28.2)	0.432	21.8 (17.1–27.3)	20.7 (16.5–26.3)	0.059
Fish and shellfish	29.0 (15.1–51.9)	30.7 (16.3–53.7)	0.236	32.3 (16.4–55.8)	27.3 (14.1–46.9)	0.001
Red and processed meat	74.4 (46.6–108.6)	75.1 (46.4–113.4)	0.604	84.5 (53.8–121.7)	73.9 (46.2–103.9)	<0.001
Total dairy	283.9 (165.0–451.4)	300.5 (197.7–458.4)	0.100	276.2 (155.1–459.2)	284.3 (186.0–479.3)	0.149
Milk	160.0 (34.3–294.6)	182.6 (59.4–347.2)	0.054	160.0 (35.5–301.4)	191.2 (53.5–311.2)	0.054
Fermented dairy	73.0 (33.9–138.6)	71.4 (34.6–143.0)	0.877	65.7 (27.6–129.8)	62.9 (27.6–127.8)	1.000
Sum of plasma phospholipid 15:0 and 17:0, mol%	0.63 (0.13)	0.64 (0.12)	0.167	0.57 (0.13)	0.59 (0.13)	<0.001
Plasma phospholipid 15:0, mol%	0.21 (0.06)	0.22 (0.06)	0.193	0.20 (0.06)	0.21 (0.06)	0.002
Plasma phospholipid 17:0, mol%	0.41 (0.09)	0.42 (0.08)	0.273	0.37 (0.09)	0.39 (0.09)	0.002

Data are mean (SD), %, or median (IQR) unless otherwise indicated

^a^*p* value for the comparison between GAD65Ab positive and negative subcohort participants

^b^*p* value for the comparison between GAD65Ab positive and negative cases

^c^Of which 669 (4.6%) developed diabetes during follow-up

^d^Of which 23 (7.7%) developed diabetes during follow-up

^e^Waist circumference measurements missing for Umeå centre (*n*=1620) and for some individuals in other centres (*n*=90)

^f^Family history information available for 49.2% of the subcohort (47.5% for GAD65Ab positive) and 49.0% (53.7% for GAD65Ab positive) of incident diabetes cases

### Correlations between OCFA and dietary factors

The sum of plasma phospholipid 15:0 and 17:0 was significantly but weakly correlated with total dairy (*r*=0.19 [95% CI 0.17, 0.21]), milk (*r*=0.13 [95% CI 0.11, 0.14]) and fermented dairy (*r*=0.13 [95% CI 0.12, 0.15]) intakes (ESM Table 2). For 15:0, the strongest correlations were seen with total dairy (*r*=0.23 [95% CI 0.21, 0.24]), fermented dairy (*r*=0.24 [95% CI 0.22, 0.25]) and fish and shellfish (*r*=−0.22 [95% CI −0.23, −0.20]) intakes. The strongest correlations for 17:0 were with fruit (*r*=0.21 [95% CI 0.20, 0.23]) and vegetables (*r*=0.16 [95% CI 0.15, 0.18]) followed by total dairy (*r*=0.13 [95% CI 0.12, 0.15]), milk (*r*=0.12 [95% CI 0.11, 0.14]) and total dietary fibre (*r*=0.10 [95% CI 0.08, 0.12]) intakes. The median intakes of dairy products and other dietary factors across concentrations of plasma phospholipid 15:0 or 17:0 are presented in ESM Table 3. The groups with the highest concentrations of these OCFA had the highest intakes of total dairy products. In addition, those with the highest 17:0 concentrations had the highest intakes of fruit, vegetables and total dietary fibre.

### Incidence of diabetes in relation to GAD65Ab status, OCFA and dairy intake

Being positive for GAD65Ab at baseline was associated with a higher incidence of diabetes (HR 1.83 [95% CI 1.51, 2.22]), and the association was even more pronounced in those with high GAD65Ab positivity (HR 3.05 [95% CI 2.37, 3.93]) (Fig. 1). Regarding OCFA, there was an inverse association between 15:0 and/or 17:0 concentrations and diabetes incidence, both in GAD65Ab-positive individuals and GAD65Ab-negative individuals (Fig. 1, Table 2). The inverse association appeared more pronounced for 17:0 than 15:0; in GAD65Ab-negative individuals the incidence of diabetes was 55% higher per 1 SD lower 17:0 (HR 1.55 [95% CI 1.48, 1.64]) and 25% higher per 1 SD lower 15:0 (HR 1.25 [95% CI 1.20, 1.32]) (Fig. 1). In GAD65Ab-positive individuals the incidence of diabetes was 69% higher per 1 SD lower 17:0 (HR 1.69 [95% CI 1.34, 2.13]) (Fig. 1) and the HR for the lowest vs highest tertile was estimated to be 1.97 (95% CI 1.25, 3.11) (Table 2). Associations were weaker in individuals with high GAD65Ab positivity (Fig. 1, Table 2); the lowest vs highest tertile of 17:0 concentration was associated with higher diabetes incidence, whereas no significant associations were observed with 15:0 or the sum of the OCFA. Intakes of total dairy products, milk or fermented dairy products were not associated with the incidence of diabetes in either GAD65Ab-negative individuals (HR 0.98 [95% CI 0.94, 1.02] per 1 SD lower total dairy intake) or GAD65Ab-positive individuals (HR 0.97 [95% CI 0.79, 1.18] per 1 SD lower total dairy intake) (Fig. 1).

**Table 2:** Interaction between GAD65Ab positivity and the lowest tertile of plasma phospholipid 15:0 and/or 17:0 on the incidence of adult-onset diabetes

GAD65Ab status	High OFCA concentration^a^		Moderate OFCA concentration^b^		Low OFCA concentration^c^		Lowest vs highest tertile of OCFA concentrations within strata of GAD65Ab
	*N* cases/non-cases	HR (95% CI)	*N* cases/non-cases	HR (95% CI)	*N* cases/non-cases	HR (95% CI)	HR (95% CI)
Sum of 15:0 and 17:0							
Negative	1755/4679	1.00 (reference)	3231/4565	1.74 (1.60, 1.90)	5749/4656	2.52 (2.30, 2.75)	2.53 (2.32, 2.76)
Positive	90/102	2.66 (1.97, 3.60)	124/101	2.79 (1.99, 3.91)	175/71	4.63 (3.33, 6.43)^d^	1.74 (1.12, 2.69)
Positive-low	22/65	1.15 (0.69, 1.93)	52/61	1.77 (1.10, 2.84)	92/44	3.49 (2.27, 5.37)	3.06 (1.58, 5.94)
Positive-high	68/37	4.64 (3.12, 6.91)	72/40	4.74 (3.07, 7.34)	83/27	6.90 (4.28, 11.11)^d^	1.49 (0.81, 2.75)
15:0							
Negative	2231/4561	1.00 (reference)	3658/4643	1.36 (1.25, 1.48)	4844/4696	1.65 (1.50, 1.80)	1.66 (1.51, 1.82)
Positive	108/98	2.31 (1.60, 3.32)	122/90	2.57 (1.87, 3.52)	159/86	2.67 (1.95, 3.66)^d^	1.16 (0.72, 1.86)
Positive-low	28/60	1.01 (0.55, 1.85)	54/55	1.68 (1.09, 2.60)	84/55	2.09 (1.38, 3.17)	2.10 (1.01, 4.35)
Positive-high	80/38	4.21 (2.80, 6.34)	68/35	4.34 (2.82, 6.68)	75/31	3.65 (2.31, 5.76)^d^	0.88 (0.48, 1.61)
17:0							
Negative	1738/4434	1.00 (reference)	2710/4483	1.41 (1.29, 1.55)	6284/4983	2.61 (2.39, 2.85)	2.62 (2.39, 2.86)
Positive	88/97	2.27 (1.64, 3.14)	98/93	2.75 (1.99, 3.79)	203/84	4.48 (3.20, 6.26)^d^	1.97 (1.25, 3.11)
Positive-low	27/62	1.16 (0.68, 1.95)	41/58	1.81 (1.16, 2.82)	98/50	3.07 (1.96, 4.80)	2.68 (1.35, 5.29)
Positive-high	61/35	3.98 (2.64, 5.99)	57/35	4.38 (2.72, 7.04)	105/34	7.51 (4.83, 11.69)^d^	1.89 (1.04, 3.42)

HRs adjusted for age (underlying time scale), centre (stratified baseline hazard), sex, education level, smoking status, physical activity, BMI, total energy intake and intake of alcohol, fruits, vegetables, cereal and cereal products, fish and shellfish, and red and processed meat (g/day)

^a^High concentration (mol%): ≥0.68 for sum of 15:0 and 17:0; ≥0.24 for 15:0; ≥0.45 for 17:0

^b^Moderate concentration (mol%): 0.58 to <0.68 for sum of 15:0 and 17:0; 0.19 to <0.24 for 15:0; 0.39 to <0.45 for 17:0

^c^Low concentration (mol%): >0 to <0.58 for sum of 15:0 and 17:0; >0 to <0.19 for 15:0; >0 to <0.39 for 17:0

^d^Measure of interaction on additive scale. AP (95% CI) for the combination of GAD65Ab positivity and the lowest tertile of 15:0 and 17:0 = 0.10 (−0.08, 0.28), *p*=0.291; 15:0 = −0.12 (−0.36, 0.13), *p*=0.349; and 17:0= 0.13 (−0.03, 0.30), *p*=0.113. AP (95% CI) for the combination of high GAD65Ab positivity and the lowest tertile of 15:0 and 17:0 = 0.13 (−0.12, 0.38), *p*=0.301; 15:0 = −0.36 (−0.77, 0.05), *p*=0.088; and 17:0 = 0.25 (0.05, 0.45), *p*=0.016. Measure of interaction on multiplicative scale: for the combination of GAD65Ab positivity and the lowest tertile of 15:0 and 17:0, *p*=0.092; 15:0, *p*=0.132; and 17:0, *p*=0.227. Measure of interaction on multiplicative scale: for the combination of high GAD65Ab positivity and the lowest tertile of 15:0 and 17:0, *p*=0.092; 15:0, *p*=0.041; and 17:0, *p*=0.283

### Interaction between GAD65Ab status and OCFA

**Fig. 2:**
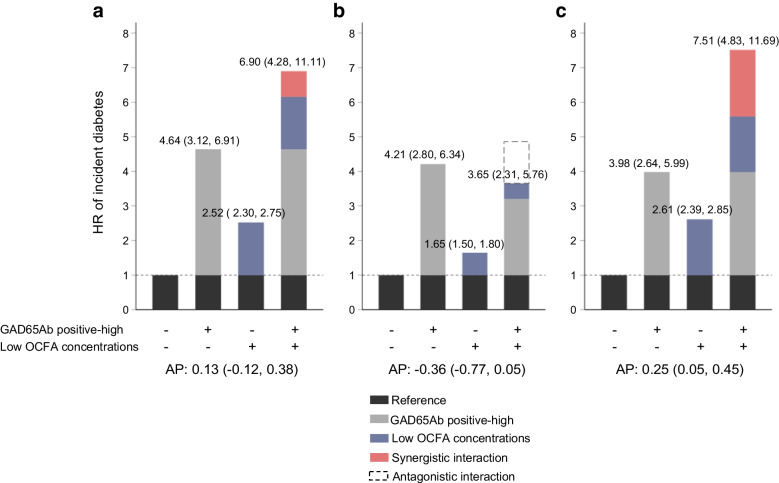
HR (95% CI) of incident diabetes in relation to high GAD65Ab positivity (indicated by + −), the lowest tertile of concentrations of plasma phospholipid 15:0 plus 17:0 (**a**), 15:0 alone (**b**) or 17:0 alone (**c**) (indicated by − +), or the combination of high GAD65Ab positivity and the lowest tertile of plasma phospholipid 15:0 and/or 17:0 concentrations (indicated by + +). The reference group is the combination of GAD65Ab negativity and the highest tertile of plasma phospholipid 15:0 and/or 17:0 concentrations (indicated by − −). Analyses were adjusted for age (underlying time scale), centre (stratified baseline hazard), sex, education level, smoking status, physical activity, BMI, total energy intake and intake of alcohol, fruits, vegetables, cereal and cereal products, fish and shellfish, and red and processed meat (g/day)

Analyses of the incidence of diabetes in relation to the combined exposure to GAD65Ab positivity and tertiles of OCFA revealed the highest HR in individuals with GAD65Ab positivity and low concentrations of 17:0 (HR 4.48 [95% CI 3.20, 6.26]) compared with those with GAD65Ab negativity and high concentrations of 17:0 (Table 2). However, there was no additive or multiplicative interaction between GAD65Ab positivity and OCFA concentrations (Table 2). Separate analyses for the combination of high GAD65Ab positivity and the lowest tertile of OCFA revealed a high incidence in individuals with low concentrations of 17:0 (HR 7.51 [95% CI 4.83, 11.69]), with evidence of additive (synergistic) interaction and AP estimated at 0.25 (95% CI 0.05, 0.45) (Table 2, Fig. 2). This indicates that 25% of the risk among the doubly exposed could be attributed to this interaction. There was no significant additive interaction between high GAD65Ab positivity and 15:0 (Table 2, Fig. 2). Multiplicative interaction was only observed between high GAD65Ab positivity and 15:0 (Table 2).

### Sensitivity analyses

The inverse associations between plasma phospholipid OCFA and diabetes remained largely unchanged in sensitivity analyses among GAD65Ab-positive individuals (ESM Fig. 2). Estimates for the combined exposure to high GAD65Ab positivity and the lowest tertile of 17:0 were also similar across the different sensitivity analyses (ESM Fig 3). Associations were weaker in the subsample with available information on family history of diabetes (49%). However, this does not seem to reflect confounding since the HRs estimated in this subsample were unaffected by adjustment for family history of diabetes (e.g. HR of diabetes per 1 SD lower 15:0 in GAD65Ab-positive individuals was 1.23 [95% CI 0.85, 1.79] before adjustment and 1.23 [95% CI 0.90, 1.69] after adjustment). The association between 15:0 and diabetes was attenuated after adjustment for 17:0 concentrations (ESM Fig. 2).

### Post hoc analyses

Neither fruit nor vegetable intake was associated with progression from GAD65Ab positivity to adult-onset diabetes (ESM Fig. 4). The corresponding HRs per 1 SD lower intake of these foods were 1.11 (95% CI 0.89, 1.39) and 1.11 (95% CI 0.91, 1.35), respectively. Low dietary fibre intake was associated with a higher risk of progressing from GAD65Ab positivity to diabetes (HR 1.27 [95% CI 1.02, 1.56]), while the association was weaker among individuals with high GAD65Ab levels (HR 1.18 [95% CI 0.83, 1.67]). None of these dietary items was associated with diabetes incidence among GAD65Ab-negative individuals (ESM Fig. 4).

## Discussion

### Main findings

We investigated whether plasma phospholipid concentrations of 15:0 and 17:0 or intake of dairy products were associated with progression to diabetes in adults with islet autoimmunity. The study results suggest that low 15:0 and 17:0 concentrations in conjunction with GAD65Ab may convey a higher risk for developing diabetes, as people who tested positive for GAD65Ab had a greater chance of acquiring diabetes over an 11 year period if they had low concentrations of these OCFA, particularly 17:0. Furthermore, there was an additive interaction between high levels of autoantibodies and low concentrations of 17:0, suggesting that increased 17:0 may be especially beneficial for people who have more pronounced autoimmunity. On the contrary, intake of dairy products was not related to the risk of progressing to diabetes in people with islet autoimmunity. These findings add to the limited understanding of potentially modifiable risk factors for adult-onset autoimmune diabetes.

### Findings in relation to previous studies

Our findings suggest that low concentrations of 15:0, and particularly 17:0, may increase the risk of progressing to diabetes in individuals with islet autoimmunity, while we found no support that low concentrations of these OCFA may trigger autoimmunity. Previous analyses based on EPIC-InterAct [25] and other prospective studies [6] show inverse associations between 15:0 and 17:0 concentrations and incidence of type 2 diabetes, whereas evidence is lacking with regard to type 1 diabetes. A biological mechanism underlying these associations may be connected to the potential metabolic functions of these OCFA. For example, cross-sectional evidence suggests that higher concentrations of 15:0 and 17:0 may prevent insulin resistance and preserve beta cell function [26, 27]. Moreover, cross-sectional studies have reported inverse associations between these OCFA and adipokines related to type 2 diabetes risk, such as leptin and plasminogen activator inhibitor-1 [28, 29]. Regulation of adipokine levels may also exert beneficial effects on autoimmune diabetes incidence: a study of non-obese diabetic mice found accelerated beta cell destruction after injection of leptin [30]. Furthermore, 17:0 has been inversely associated with chronic inflammation [5], which is a proposed mediator in the association between obesity and LADA [31].

Consumption of dairy products was not associated with progression from autoantibody positivity to adult-onset diabetes. This contrasts with previous findings for type 1 diabetes in children that suggested an elevated risk with a higher intake of cow’s milk products [4]. Although a biological mechanism linking dairy consumption to type 1 diabetes has yet to be discovered, it has been hypothesised that due to structural similarities with islet cell proteins, cow’s milk proteins, which are known dietary antigens, may promote autoimmune diabetes in susceptible individuals [9]. This might be particularly relevant in infancy and childhood when immune-mediated reactions to cow’s milk proteins are more common [32] and may explain why dairy intake was neither associated with GAD65Ab positivity at baseline nor with progression to adult-onset diabetes in our study.

Because OCFA 15:0 and 17:0 have been proposed as biomarkers of dairy fat [7], these findings may appear counterintuitive. Notably, both our study and earlier studies [33, 34] found modest correlations between 17:0 and dairy product intake and, indeed, the utility of 17:0 as a biomarker of dairy intake has been questioned [35]. We found the strongest correlations between 17:0 and fruit and vegetable consumption. Concentrations of 15:0, on the other hand, were more strongly linked to consumption of dairy products, particularly fermented items, consistent with previous research [33, 34]. Furthermore, it has been proposed that 15:0 and 17:0 can be synthesised endogenously from propionate, a short-chain fatty acid that is commonly produced in the intestine after the fermentation of dietary fibre [36]. A small experimental study that compared the concentrations of 15:0 and 17:0 before and after a week of supplementation with non-fermentable dietary fibre (cellulose), fermentable dietary fibre (inulin), or propionate found an increase in these OCFA in the inulin and propionate groups [37], suggesting that fermentable dietary fibre sources like fruits, vegetables, beans and oats may contribute to higher concentrations of 15:0 and 17:0. This implies that potential contributors to higher 15:0 and 17:0 concentrations other than dairy products (e.g. fermentable dietary fibre), may play a role on the incidence of autoimmune diabetes. In support hereof, we observed a higher risk of progressing from islet autoimmunity to adult-onset diabetes in relation to low dietary fibre intakes. Interestingly, the inverse association we observed with 15:0 was attenuated after adjusting for 17:0, but not vice versa. In line with this, interaction with GAD65Ab positivity on the risk of diabetes was only observed with low 17:0 and not 15:0 concentrations, indicating that a possible beneficial effect may primarily be related to 17:0.

### Strengths and limitations

Strengths of this study include the prospective case–cohort design and the large number of participants recruited across multiple centres, representing a wide range of the European population. In addition, the diabetes diagnosis was verified from more than one source, which reduced the likelihood of false-positive diagnoses. Moreover, all GAD65Ab and plasma phospholipid fatty acid analyses took place at a laboratory in Seattle and Cambridge, respectively, by staff that were blinded to the outcome status, preventing measurement variation and the introduction of detection bias. Another strength was the use of country-specific questionnaires to assess diet, thus helping to capture the diverse intakes across populations. In addition, we adjusted for several potential confounders, including demographic, lifestyle and clinical characteristics.

Limitations include potential measurement errors that may have led to residual confounding or misclassification of the exposures. Due to the prospective study design, those are not likely to be differential between cases and non-cases and hence might have attenuated the associations. It should also be noted that there was a large proportion of missing values on important covariates, such as family history of type 2 diabetes, which could only be adjusted for in subsamples. Moreover, information on autoantibody status was not available at the time of diagnosis and thus it was not possible to verify whether people with GAD65Ab positivity at baseline were still positive at the time of diagnosis and thus met the criteria of LADA. It is also possible that some individuals had undiagnosed diabetes at baseline, although this is not likely to have affected our results, which remained largely unchanged after excluding individuals that had received a diabetes diagnosis during the first 2 years or had HbA_1c_ ≥48 mmol/mol (≥6.5%) at baseline. Moreover, autoantibody status, fatty acid concentrations and dietary intakes likely changed during the follow-up but it was not possible to account for such changes as all exposures were only assessed at baseline. Furthermore, even though there was sufficient statistical power to investigate interactions between GAD65Ab and OCFA concentrations, it was not possible to assess these within each country. Still, potential variations in the baseline hazard across the participating centres were accounted for in the analyses. We observed interaction on the additive scale but not on the multiplicative scale for 17:0. Additive interaction is more relevant for our objectives since it estimates the proportion of people who would not have developed the disease if just one of the analysed factors had been present [24] (e.g. high GAD65Ab positivity but not low 17:0 concentrations). Under the assumption of causality and the lack of measurement error, this interpretation holds. However, having interaction on both the additive and multiplicative scale has been considered the strongest kind of interaction [38], therefore, our observation should be regarded with caution and be used primarily to generate hypotheses.

### Implications of the findings

Our findings on the association of high GAD65Ab levels with diabetes incidence when combined with 17:0 concentrations provide guidance for future interventions to prevent autoimmune diabetes in adults. Nonetheless, because these findings are novel and based on observational data, further evidence, especially from randomised controlled trials, is needed to test whether they are causative and generalisable to populations outside of Europe. It is also worth noting that identifying people with islet autoimmunity might be difficult because autoantibody measurements are not part of normal clinical assessment and are usually done after diabetes symptoms have arisen. However, if preventative factors for autoimmune diabetes become well-established, this may change.

In conclusion, we find that higher plasma phospholipid concentrations of the OCFA 17:0 are associated with a lower risk of progression to diabetes in individuals with islet autoimmunity. Future investigations of modifiable risk factors for autoimmune diabetes should include exposures (apart from dairy products) that could influence 17:0 concentrations.

## Supplementary Information

Below is the link to the electronic supplementary material.

Supplementary file1 (PDF 576 KB)

## Data Availability

The datasets analysed in the current study are available from the corresponding author upon reasonable request.

## Abbreviations

AP: Proportion attributable to interaction
EPIC: European Prospective Investigation into Cancer and Nutrition
GAD65Ab: Autoantibodies against GAD65
IARC: International Agency for Research on Cancer
LADA: Latent autoimmune diabetes in adults
OCFA: Odd-chain fatty acids
