# Supplementary material for: Interaction between plasma phospholipid odd-chain fatty acids and GAD65 autoantibodies on the incidence of adult-onset diabetes: the EPIC-InterAct case–cohort study
Source: Diabetologia. 2023 Jun 10;66(8):1460–71. doi: 10.1007/s00125-023-05948-x (PMC10317878; doi:10.1007/s00125-023-05948-x)
Supplement: Supplementary file 1 — Supplementary file1 (PDF 576 KB) [file 125_2023_5948_MOESM1_ESM.pdf]

SUPPLEMENTARY MATERIAL

Interaction between plasma phospholipid odd-chain fatty acids and GAD65 autoantibodies on the incidence of adult-onset diabetes: the EPIC-InterAct case–cohort study

Authors:

Anna-Maria Lampousi,[1] Sofia Carlsson,[1] Josefin E. Löfvenborg,[1,2] Natalia Cabrera-Castro,[3] María-Dolores Chirlaque,[3,4,5] Guy Fagherazzi,[6] Paul W. Franks,[7,8] Christiane S. Hampe,[9] Paula Jakszyn,[10,11] Albert Koulman,[12,13] Cecilie Kyrø,[14] Conchi Moreno-Iribas,[4,15,16] Peter M. Nilsson,[7] Salvatore Panico,[17] Keren Papier,[18] Yvonne T. van der Schouw,[19] Matthias B. Schulze,[20,21,22] Elisabete Weiderpass,[23] Raul Zamora-Ros,[10] Nita G. Forouhi,[12] Stephen J. Sharp,[12] Olov Rolandsson,[8] and Nicholas J. Wareham [12]

Correspondence to:

Anna-Maria Lampousi

Institute of Environmental Medicine, Karolinska Institute

Box 210, 171 77 Stockholm, Sweden

+46702575629

annamaria.lampousi@ki.se

ESM Table 1. Adjusted OR (95% CI) of GAD65Ab positivity or high positivity vs negativity at baseline in relation to baseline plasma phospholipid 15:0 and/or 17:0 and dairy intake in tertiles or per SD increase based on the distribution in the subcohort.

Exposure	GAD65Ab positive		GAD65Ab positive-high	
	N positive/negative	OR (95% CI)	N positive-high/negative	OR (95% CI)
**Sum of 15:0 and 17:0 (mol%)**				
Low (>0 to <0.58)	246/10,405	1.00 (reference)	110/10,405	1.00 (reference)
Moderate (0.58 to <0.68)	225/7,796	1.14 (0.94, 1.39)	112/7,796	1.17 (0.89, 1.55)
High (≥0.68)	192/6,434	1.17 (0.95, 1.46)	105/6,434	1.28 (0.94, 1.74)
Per 1 SD increase (0.13)		1.03 (0.94, 1.12)		1.04 (0.91, 1.18)
**15:0 (mol%)**				
Low (>0 to <0.19)	245/9,540	1.00 (reference)	106/9,540	1.00 (reference)
Moderate (0.19 to <0.24)	212/8,301	0.90 (0.74, 1.10)	103/8,301	0.86 (0.64, 1.15)
High (≥0.24)	206/6,792	1.03 (0.83, 1.28)	118/6,792	1.04 (0.77, 1.41)
Per 1 SD increase (0.06)		1.03 (0.94, 1.13)		1.02 (0.90, 1.15)
**17:0 (mol%)**				
Low (>0 to <0.39)	287/11,267	1.00 (reference)	139/11,267	1.00 (reference)
Moderate (0.39 to <0.45)	191/7,193	0.97 (0.80, 1.18)	92/7,193	0.93 (0.70, 1.22)
High (≥0.45)	185/6,172	1.12 (0.90, 1.38)	96/6,172	1.23 (0.91, 1.65)
Per 1 SD increase (0.09)		1.02 (0.93, 1.11)		1.04 (0.92, 1.18)
**Total dairy (g/day)**				
Low (<207.4)	193/8,469	1.00 (reference)	85/8,469	1.00 (reference)
Moderate (207.4 to <380.8)	234 /7,971	1.22 (1.00, 1.49)	120 /7,971	1.30 (0.97, 1.74)
High (≥380.8)	236/8,195	1.15 (0.92, 1.43)	122/8,195	1.08 (0.79, 1.48)
Per 1 SD increase (239)		1.02 (0.94, 1.12)		1.01 (0.90, 1,14)
**Milk (g/day)**				
Low (<75)	191/8,313	1.00 (reference)	85/8,313	1.00 (reference)
Moderate (75 to <235.7)	215/8,035	1.04 (0.85, 1.28)	111/8,035	1.19 (0.88, 1.59)
High (≥235.7)	257/8,287	1.16 (0.93, 1.44)	131/8,287	1.14 (0.83, 1.56)
Per 1 SD increase (211)		1.03 (0.95, 1.12)		1.01 (0.90, 1.13)
**Fermented dairy (g/day)**				
Low (<45)	229/8,608	1.00 (reference)	103/8,608	1.00 (reference)
Moderate (45 to <111.4)	216/8,084	1.03 (0.85, 1.26)	110/8,084	1.16 (0.87, 1.55)
High (≥111.4)	218/7,943	1.05 (0.85, 1.30)	114/7,943	1.18 (0.87, 1.60)
Per 1 SD increase (95)		0.98 (0.90, 1.07)		1.02 (0.91, 1.14)

ORs adjusted for age, centre, sex, education level, smoking status, physical activity, BMI, total energy intake and intake of alcohol, fruits, vegetables, cereal and cereal products, fish and shellfish, and red and processed meat (g/day)

ESM Table 2. Spearman rank correlation matrix for plasma phospholipid 15:0 and/or 17:0 and different dietary factors in the subcohort

	**15:0 +17:0**	**15:0**	**17:0**	**Total dairy**	**Milk**	**Fermented dairy**	**Fruit**	**Vegetables**	**Cereal**	**Total dietary fibre**	**Fish & shellfish**	**Red & processed meat**
**15:0 +17:0**	1											
**15:0**	0.799	1										
**17:0**	0.900	0.500	1									
**Total dairy**	0.190	0.225	0.132	1								
**Milk**	0.127	0.097	0.122	0.840	1							
**Fermented dairy**	0.134	0.235	0.049	0.398	-0.029	1						
**Fruit**	0.156	0.031	0.214	0.027	0.030	0.061	1					
**Vegetables**	0.085	-0.054	0.163	0.030	0.035	0.011[a]	0.357	1				
**Cereal**	-0.033	-0.042	-0.011[a]	-0.031	-0.040	0.061	0.088	0.082	1			
**Total dietary fibre**	0.071	0.005[a]	0.101	0.105	0.050	0.122	0.483	0.466	0.547	1		
**Fish & shellfish**	-0.047	-0.218	0.079	0.016[a]	0.080	-0.068	0.170	0.356	0.080	0.219	1	
**Red & processed meat**	-0.173	-0.168	-0.145	-0.034	-0.025	-0.046	-0.126	0.013[a]	0.187	0.161	0.128	1

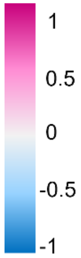

[a] p value >0.05

ESM Table 3. Median (IQR) dietary intakes by tertile of concentration of plasma phospholipid 15:0 and 17:0 among the subcohort and incident diabetes cases

**Median (IQR) g/day**	**15:0 (mol%)**			**17:0 (mol%)**		
	Low (>0 to <0.19)	*Moderate (0.19 to <0.24)*	*High (≥0.24)*	*Low (>0 to <0.39)*	*Moderate (0.39 to <0.45)*	*High (≥0.45)*
	**Subcohort**					
n	5,116	4,977	4,773	5,450	4,752	4,664
Fruit	188 (93, 325)	192 (105, 317)	200 (114, 310)	156 (84, 269)	195 (108, 310)	243 (135, 374)
Vegetables	166 (104, 258)	149 (98, 226)	152 (102, 226)	141 (92, 211)	149 (98, 230)	185 (118, 273)
Cereal and cereal products	202 (142, 279)	199 (142, 274)	191 (137, 266)	200 (141, 275)	196 (141, 273)	197 (139, 273)
Total dietary fibre	22 (17, 28)	22 (17, 27)	22 (18, 27)	21 (17, 26)	22 (18, 27)	23 (18, 28)
Fish and shellfish	37 (19, 65)	30 (16, 50)	22 (11, 40)	28 (14, 51)	27 (14, 47)	34 (17, 58)
Red and processed meat	85 (52, 124)	74 (47, 105)	66 (42, 97)	84 (52, 122)	72 (45, 104)	67 (43, 99)
Total dairy	233 (119, 366)	301 (182, 466)	331 (203, 517)	253 (128, 420)	297 (173, 469)	309 (201, 466)
Milk	149 (23, 254)	171 (43, 314)	169 (44, 328)	135 (19, 270)	164 (42, 300)	193 (67, 319)
Fermented dairy	52 (20, 107)	79 (38, 147)	92 (48, 159)	67 (30, 129)	77 (37, 143)	77 (34, 142)
	**Incident diabetes cases**					
n	4,134	3,331	3,659	4,157	3,452	3,515
Fruit	180 (86, 321)	181 (101, 308)	188 (102, 303)	154 (79, 269)	181 (98, 301)	221 (118, 365)
Vegetables	159 (98, 253)	145 (93, 224)	145 (95, 223)	138 (89, 213)	143 (93, 219)	175 (108, 266)
Cereal and cereal products	203 (143, 281)	195 (135, 269)	194 (136, 268)	198 (138, 271)	197 (139, 272)	199 (137, 277)
Total dietary fibre	22 (17, 28)	21 (17, 27)	22 (17, 27)	21 (16, 26)	22 (17, 27)	23 (18, 28)
Fish and shellfish	38 (19, 67)	31 (16, 53)	26 (14, 47)	31 (16, 53)	30 (15, 52)	36 (18, 63)
Red and processed meat	93 (59, 134)	83 (53, 120)	76 (49, 110)	91 (59, 130)	81 (52, 121)	78 (49, 113)
Total dairy	220 (106, 360)	294 (175, 483)	342 (202, 536)	247 (124, 422)	287 (172, 470)	309 (181, 486)
Milk	141 (20, 248)	175 (45, 325)	192 (54, 391)	137 (20, 290)	169 (45, 307)	192 (62, 341)
Fermented dairy	47 (17, 103)	72 (34, 136)	82 (39, 153)	62 (25, 122)	69 (29, 134)	68 (29, 136)

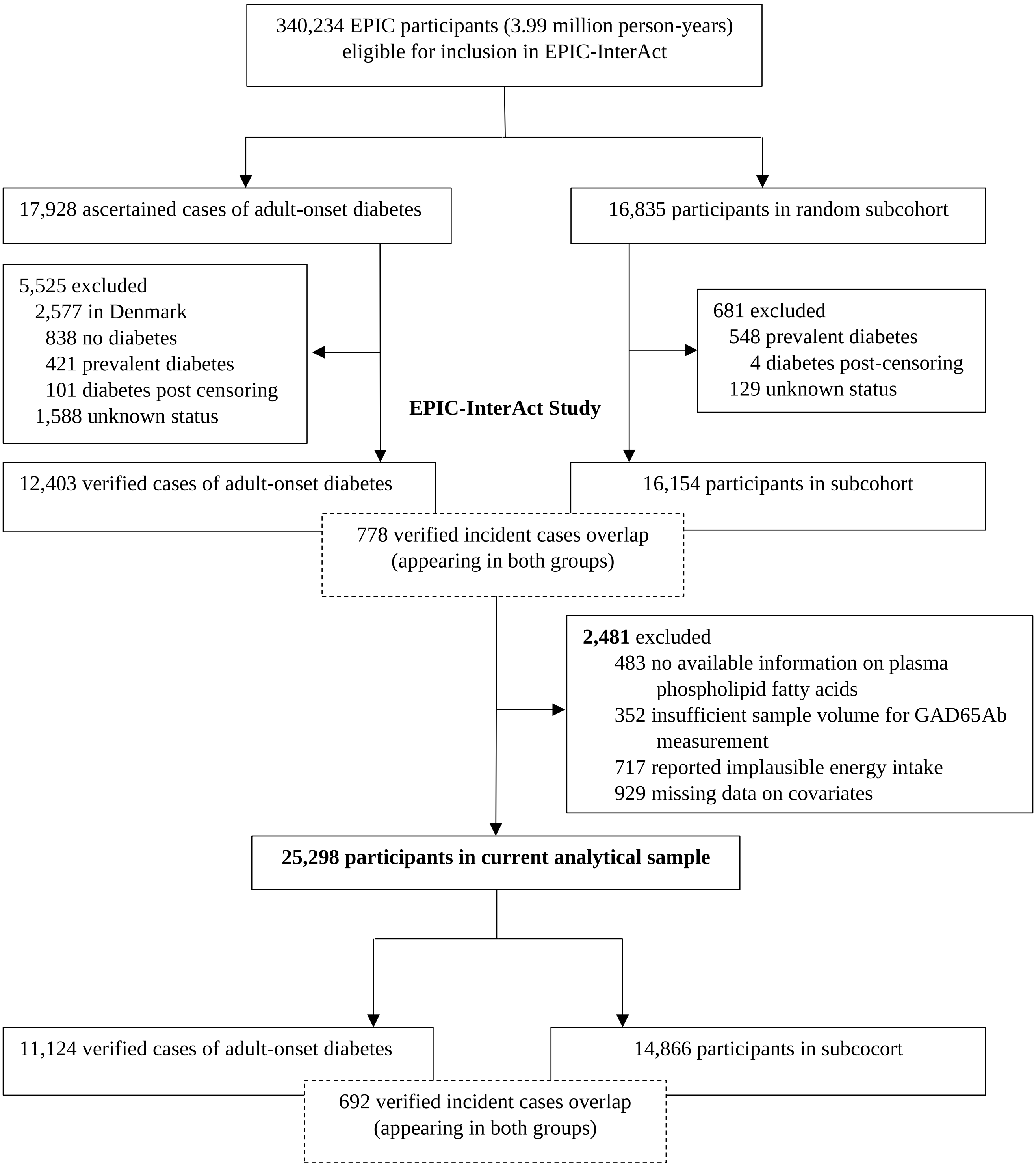

ESM Figure 1. EPIC-InterAct case-cohort study design and flowchart of study participants

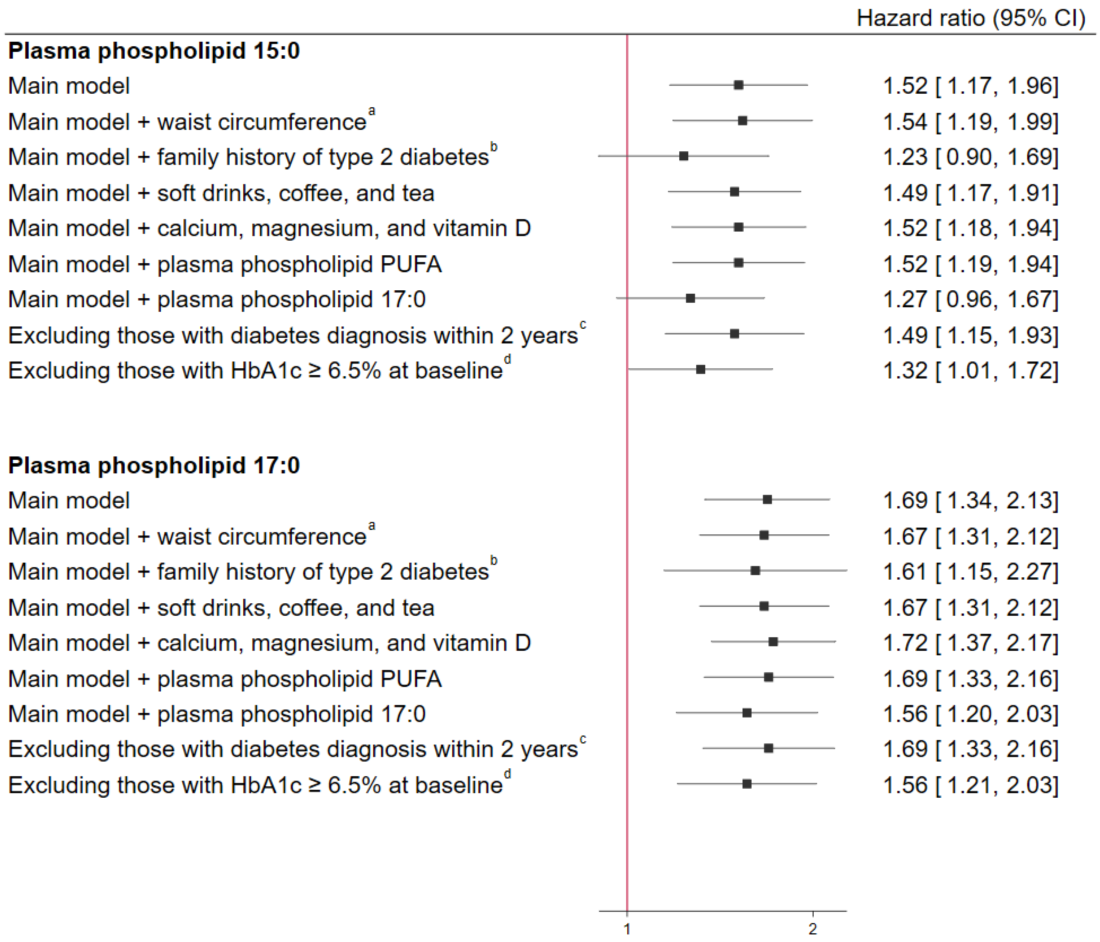

ESM Figure 2. HRs (95% CI) of incident diabetes in relation to 1 SD lower plasma phospholipid 15:0 or 17:0 among GAD65Ab positive individuals (n=663), based on different sensitivity analyses. Main model: adjusted for age (underlying time scale), centre (stratified baseline hazard), sex, education level, smoking status, physical activity, BMI, total energy intake and intake of alcohol, fruits, vegetables, cereal and cereal products, fish and shellfish, and red and processed meat (g/day). PUFA: polyunsaturated fatty acids; [a]Waist circumference measurements missing for n=57 individuals; [b]Family history information missing for n=324 individuals; [c]n=42 excluded individuals; [d]n=80 excluded individuals.

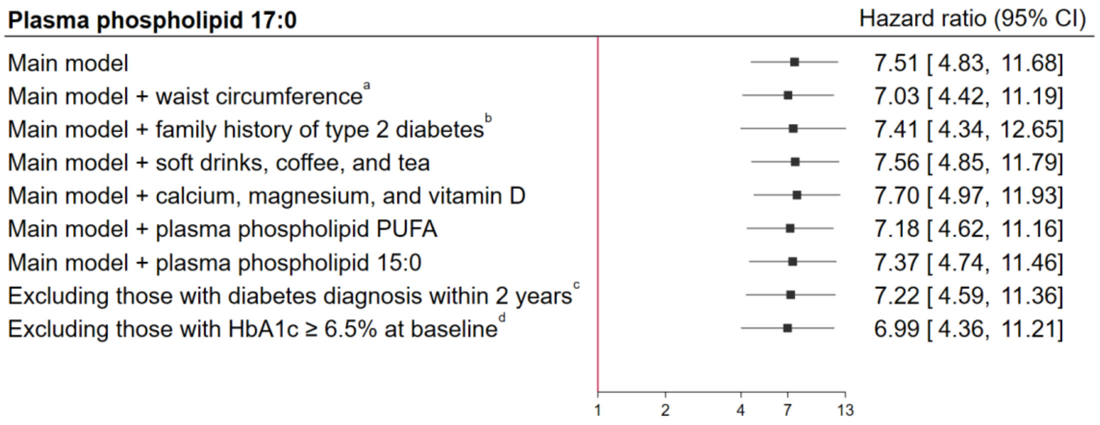

ESM Figure 3. HRs (95% CI) of incident diabetes for the combination of high GAD65Ab positivity and the lowest tertile of plasma phospholipid 17:0 concentrations, based on different sensitivity analyses. The reference group is the combination of GAD65Ab negativity and the highest tertile of plasma phospholipid 17:0 concentrations. Main model: adjusted for age (underlying time scale), centre (stratified baseline hazard), sex, education level, smoking status, physical activity, BMI, total energy intake and intake of alcohol, fruits, vegetables, cereal and cereal products, fish and shellfish, and red and processed meat (g/day). PUFA: polyunsaturated fatty acids; [a]Waist circumference measurements missing for Umeå centre (n=1,620) and for some individuals in other centres (n=90); [b]Family history information missing for n=12,832 individuals; [c]n=1,010 excluded individuals; [d]n=2,106 excluded individuals.

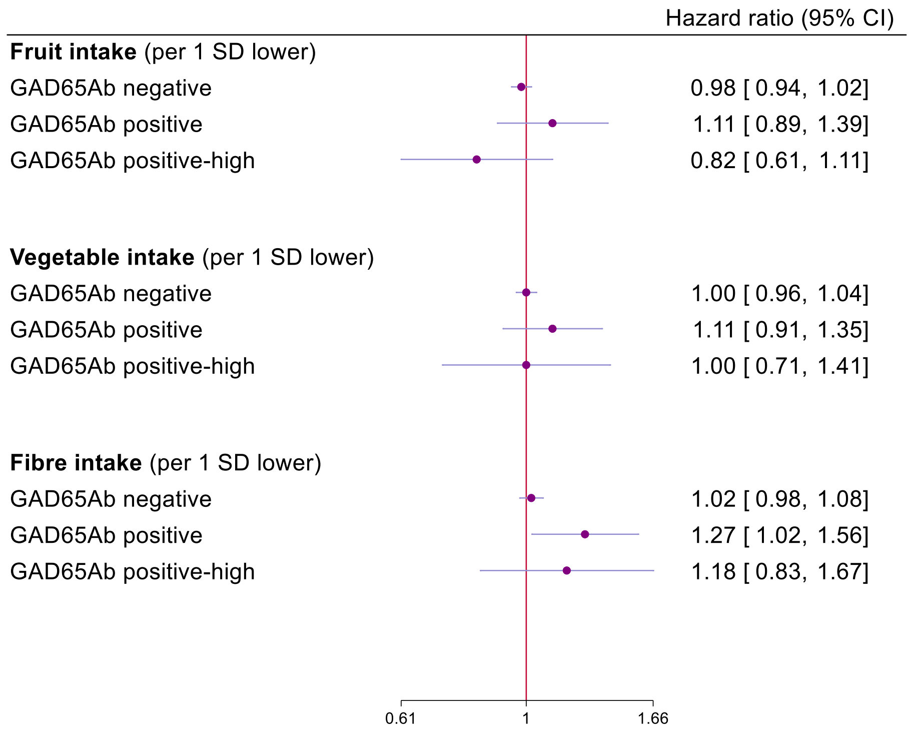

ESM Figure 4. HRs (95% CI) of incident diabetes per 1 SD lower fruit, vegetable, and total dietary fibre intakes, stratified by GAD65Ab status. HRs were adjusted for age (underlying time scale), centre (stratified baseline hazard), sex, education level, smoking status, physical activity, BMI, total energy intake and intake of alcohol, dairy products, cereal and cereal products, fish and shellfish, and red and processed meat (g/day). The analyses of fruit and vegetables were mutually adjusted.
